# Preliminary Study on Treatment Outcomes and Prednisolone Tapering after Marine Lipid Extract EAB-277 Supplementation in Dogs with Immune-Mediated Hemolytic Anemia

**DOI:** 10.3390/vetsci10070425

**Published:** 2023-06-30

**Authors:** Raktham Mektrirat, Peerawit Chongrattanameteekul, Nattanon Pureeroj, Metina Duangboon, Jarunee Loythong, Natakorn Wiset, Sineenart Chantarachart, Chompunut Lumsangkul, Wanpitak Pongkan

**Affiliations:** 1 Department of Veterinary Biosciences and Veterinary Public Health, Faculty of Veterinary Medicine, Chiang Mai University, Chiang Mai 50100, Thailand; raktham.m@cmu.ac.th (R.M.); peerawit_ch@cmu.ac.th (P.C.); natthanon_pureeroj@cmu.ac.th (N.P.); maytina_duangboon@cmu.ac.th (M.D.); 2 Research Center for Veterinary Biosciences and Veterinary Public Health, Faculty of Veterinary Medicine, Chiang Mai University, Chiang Mai 50100, Thailand; 3 Center of Excellence in Pharmaceutical Nanotechnology, Faculty of Pharmacy, Chiang Mai University, Chiang Mai 50200, Thailand; 4 Small Animal Hospital, Faculty of Veterinary Medicine, Chiang Mai University, Chiang Mai 50200, Thailand; jarunee.loy@cmu.ac.th (J.L.); natakorn_w@cmu.ac.th (N.W.); sineenart.ch@cmu.ac.th (S.C.); 5 Department of Animal and Aquatic Sciences, Faculty of Agriculture, Chiang Mai University, Chiang Mai 50200, Thailand; chompunut.lum@cmu.ac.th; 6 Multidisciplinary Research Institute, Chiang Mai University, Chiang Mai 50200, Thailand

**Keywords:** anti-inflammation, canine, immune-mediated hemolytic anemia, nutraceutical, polyunsaturated fatty acid, prednisolone

## Abstract

**Simple Summary:**

Immune-mediated hemolytic anemia (IMHA) in dogs is a common autoimmune disease which is accompanied with a high death rate and therapeutic challenges. A natural anti-inflammatory nutraceutical product, EAB-277, is derived from marine lipids. Unfortunately, the effects of EAB-277 in IMHA dogs has rarely been investigated. The objective of this study is to assess the clinical effects of EAB-277 and prednisolone dose-tapering for supplemental therapy in IMHA dogs. The findings provide evidence that standard therapy combined with EAB-277 can improve hematological and blood chemistry profiles, resulting in a higher survival rate in IMHA dogs. Furthermore, EAB-277 supplementation can reduce prednisolone dosage tapering and improve the quality of life of IMHA dogs. However, a longer-term study with a larger sample size is necessary to corroborate these findings. As a result, marine EAB-277 is a promising alternative to existing medication for IMHA. Since the nutraceuticals have been utilized not only for nutrition but also as supplemental therapy for the treatment of a wide range of illnesses, such as minimizing the adverse effects of immunosuppressive therapy with steroids.

**Abstract:**

Immune-mediated hemolytic anemia (IMHA) is a common autoimmune disorder in dogs with a high fatality rate and it remains a therapeutic challenge. The marine lipid extract, EAB-277, is a natural anti-inflammatory nutraceutical product. However, the effects of EAB-277 in IMHA dogs has rarely been investigated. The objective of this study is to assess the clinical effects of EAB-277 and prednisolone dose-tapering for supplemental therapy in IMHA dogs. Prednisolone was given to 18 anemic IMHA dogs according to a standard regimen. Six dogs were supplementally treated with EAB-277 for 28 days and the remaining twelve dogs were a control group of untreated supplementations. The results demonstrate that the supplement group showed slightly better survival rates (66.7 ± 19.2%) than the control group (16.7 ± 0.7%), but the difference was not statistically significant (*p* = 0.408). When compared to pre-therapy, the supplement group’s blood profiles improved (*p* < 0.05). The EAB-277 treated group showed a moderate decrease in the incidence rate (4.20 times) of prednisolone tapering compared to the control group. The dosage reduction of prednisolone in supplement group was more than that in the control group (*p* < 0.0001). Our results suggest that EAB-277 supplementation may enhance clinical outcomes and lessen prednisolone dose-tapering in canine IMHA therapy.

## 1. Introduction

Immune-mediated hemolytic anemia (IMHA) is a common autoimmune disorder associated with a considerably high complication and fatality rate in dogs [1]. In the past few years, the incidence of canine IMHA has been reported as approximately 1 in 10,000 in certain breeds [2]. Primary idiopathic IMHA develops spontaneously in dogs and is associated with certain breeds, whereas the induction of secondary IMHA has also been reported after vaccinations, antibiotic administrations, neoplasm, and infections [3]. The immunopathogenesis of IMHA is believed to be linked to anti-erythrocyte autoantibodies [4]. The systemic inflammatory response and cytokine dysregulation has been documented in dogs with IMHA [5,6]. In addition, this life-threatening disorder is characterized by an auto-inflammatory process, leading to the severe intravascular and extravascular immune-mediated destruction of erythrocytes.

IMHA remains a therapeutic challenge in veterinary practice. Several papers reporting successful chemotherapeutic clinical trials of the immunosuppressive drugs in the treatment of canine IMHA have thus been published [7,8]. Corticosteroids are generally used as a first-line immunosuppressive therapy for canine IMHA by non-specific immune suppressive effects for the control of autoimmunity targeting of erythrocyte antigens [8,9]. However, the steroid-medication-related adverse drug reactions resulting from immunosuppressive use and the prolonged therapeutic period are well documented [10]. In an endeavor to taper off the glucocorticoid, a therapeutic regimen with safety and efficacy profiles of routine anti-inflammatory supplementation combined with immunosuppressive glucocorticoids in canine patients with IMHA was not strongly recommended.

A marine lipid extract, EAB-277, which is a potent blend of two marine lipid concentrates including the lipid fraction extracted from greenshell mussels (*Perna canaliculus*), and a high-phospholipid extract from krill (*Euphausia superba*). This product was developed from PCSO-524, the patented dietary supplement, which contains various long-chain polyunsaturated fatty acids (PUFA) including eicosapentaenoic acid (EPA), docosahexaenoic acid (DHA), and eicosatetraenoic acid (ETA) [11]. The unique combination of omega 3 PUFAs affecting lipoxygenase (LOX) and cyclooxygenase (COX) pathways on reducing the production of leukotrienes and prostaglandins has been demonstrated [12]. Increasingly, the functional fatty acids from marine lipid extract are a significant bioresource and have various veterinary applications including the treatment of osteoarthritis, degenerative spinal diseases, and patellar luxation repair [13,14,15]. Unfortunately, this dietary supplement has never been evaluated for use with systemic inflammatory reactions, particularly IMHA.

Therefore, the primary aim of the present study was to evaluate the efficacy of supplementation with a marine lipid extract EAB-277 combined with standard immunosuppressive therapy on treatment outcomes in dogs with IMHA. We hypothesized that supplementation with the marine lipid extract, EAB-277, would significantly reduce prednisolone dosage, improve clinical parameters, and increase the survival rate in IMHA patient dogs compared to standard therapy.

## 2. Materials and Methods

### 2.1. Chemical Analysis of the Marine Lipid Extract EAB-277

The extraction methods of greenshell mussels and krill were first stabilized and freeze-dried. The natural ingredients were isolated by the supercritical fluid extractor using a supercritical carbon dioxide (SC-CO_2_). Reference quantification of major lipid composition was conducted by the commercial food chemical Laboratory, using the Official Methods of Analysis (OMA) program of Association of Official Analytical Chemists (AOAC), including AOAC 948.15 OMA and AOAC 963.22 OMA online.

### 2.2. Animal and Ethical Approval

The clinical study was a cross-sectional investigation that included owned dogs at the Small Animal Hospital, Faculty of Veterinary Medicine, Chiang Mai University. The owners of the animals participating were required to provide informed consent before procedure was performed. Eighteen client-owned dogs with IMHA were enrolled. The dogs remained under the care of their owners during the study. The experiment followed the protocols authorized by the Animal Ethics Committee of the Faculty of Veterinary Medicine, Chiang Mai University. (Ethic Permit No. S15/2563).

### 2.3. Criteria for Case Selection

All potential study dogs underwent a clinical screening including medical history, physical examination, hematological examination, serum biochemical analysis, rapid osmotic fragility tests (ROFTs). The saline agglutination test was used to determine the presence of true erythrocyte auto-agglutination. Direct antiglobulin tests (DAT) were conducted to establish a diagnosis of IMHA. Eligibility for inclusion included dogs with anemia (hematocrit, Hct < 35% and hemoglobin, Hb < 11.9 g/dL) and hyperbilirubinemia. They must also have had one or more of the following criteria: the presence of autoagglutination and a positive saline agglutination test; the presence of significant numbers of spherocytes on a blood smear (8–20 spherocytes per 100× magnification field); the positive ROFT and the positive DAT [16]. Dogs with IMHA were excluded if examinations revealed an underlying cause for hemolytic anemia. Furthermore, any dogs who had received any blood product or immunosuppressants for more than 48 h were also excluded from the clinical study.

### 2.4. Drugs and Dosing Procedures

The dogs with IMHA who met the inclusion criteria were randomly assigned to either glucocorticoid alone (control group, *n* = 12), or glucocorticoids and EAB-277 (supplement group, *n* = 6). All enrolled dogs, in both the control and supplement groups, were treated with immunosuppressive dosages of prednisolone (2–4 mg/kg PO q12 h) [10]. The dogs with IMHA in the supplement group, designed as an open-labeled test, were treated with EAB-277 at initial dosage of 100 mg/10 kg body weight PO once daily for 14 days followed by maintenance dosage of 50 mg/10 kg body weight PO once daily for 14 days.

### 2.5. Clinical Monitoring and Data Collection

The clinical data were obtained from the medical records of selected IMHA animals. All animals were closely observed periodically at 0, 7, 14, 21, and 28 days for any clinical outcomes and their survival performance. For serum biochemical analysis, blood was drawn from the cephalic vein, and the sera were separated via centrifugation at 2200× *g* for 15 min (Kokusan H-19α, Kokusan, Saitama, Japan), after stabilization at room temperature for 30 min. Blood biochemical parameters were measured using an automated BX-3010 analyzer (Sysmex, Kobe, Japan). Canine ethylenediamine tetraacetic acid (EDTA) whole blood was collected and the complete bloods counts were measured using an automated hematology analyzer (BC-5300 Vet, Mindray, Shenzhen, China). The in-saline auto-agglutination test was conducted by mixing of EDTA blood and 0.9% normal saline solution (NSS) on a smear slide. The agglutination of erythrocytes was observed macroscopically and microscopically [17]. The degree of spherocytosis was carried out by counting the number of spherocytes in 10 different oil immersion fields (100×), followed by calculating the mean value per HPF. The ROFT for spherocytosis was also evaluated using 0.9% and 0.55% NSS [18]. The erythrocytes were washed three times with 0.9% normal saline before the direct antiglobulin test with a commercial reagent, according to the manufacturer’s instructions (MP Biomedicals, Solon, OH, USA).

### 2.6. Calculation of Tapering Regimen

Tapering of the initial dose of prednisolone was performed when clinical improvements were observed and Hct had been stable at >30% for 2 weeks after commencing treatment. After response to treatment, the prednisolone dosage is gradually tapered by 25%. The size of daily dose was calculated after tapering the dosage. The rate of dose-tapering in dogs in the supplement group compared with that of in the control group was also analyzed.

### 2.7. Statistical Analysis

For the primary endpoint (28-day all-cause death), the standard protocol of glucocorticoid was compared with the supplementary protocol (glucocorticoid and EAB-277) via Kaplan–Meier survival analysis and the log-rank test for significance. The Shipiro–Wilk test and normal Q-Q plots were used to ensure the normal distribution in the continuous variable distribution. Descriptive statistics, including numbers, percentages, proportion, range, mean with standard deviation, and median with range of interquartile have been used to describe the data. Differences between experimental groups in hematological and blood biochemical parameters were further assessed using Student’s t test or Mann–Whitney U test. The paired *t*-test or Wilcoxon signed rank test was used to compare between pre- and post-therapy within group. The statistical significance of tapering rate for the different groups was determined from a one-tailed standard normal distribution table using the Z statistic. The statistical significance of variable differences was determined by analyzing whether the bicaudal probability of occurrences owing to chance (error type I) was less than 5% (*p* < 0.05). The R statistics (RStudio, Boston, MA, USA) was carried out. Commercial GraphPad Prism software (San Diego, CA, USA) was used for graph generation.

## 3. Results

### 3.1. Lipid Contents of EAB-277

The EAB-277 from the greenshell mussels and krill was prepared via supercritical fluid extraction. Liquid-filled and sealed gelatin capsule was the pharmaceutical dosage form. The constituents were characterized via the standard AOAC method. The lipid composition of the EAB-277 capsules was determined by total fat (97.0 g/100 g) content, which consisted mainly of unsaturated fats (77.9 g/100 g) and small amounts of saturated fats (18.2 g/100 g). The unsaturated fats were divided into mono-unsaturated fat (61.7 g/100 g) and poly-unsaturated fat (16.2 g/100 g). Interestingly, an omega 3 fatty acid was present (10.5 g/100 g). The abundant compounds of were C20:5n3 EPA, accounting for about 5.5% followed by C22:6n3 DHA 5.5%.

### 3.2. Baseline Characteristics of Patient Dog with IMHA

Eighteen dogs with IMHA were consecutively volunteered into the study. Twelve dogs were assigned to the control group and six dogs to the supplement group. No significant difference was found between the control group and the supplement group in weight, age, sex, Hct and Hb at presentation. The presence of the spherocytosis and the agglutination, as markers of IMHA were also not statistically different between the groups. The previously proposed diagnostic indicators, ROFT and DAT, were successufully comfirmed at the time of presentation (Table 1).

### 3.3. Survival Outcomes

**Figure 1:**
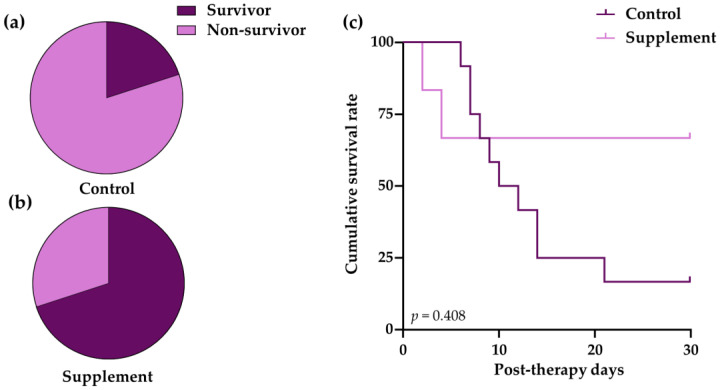
The mortality and survival analysis of IMHA dogs treated with prednisolone alone (control group) and prednisolone combined with EAB-277 (supplement group). Pie chart to show percentage mortality in (**a**) control group and (**b**) supplement group at 28 days after treatment. Fisher’s exact test was used for statistical analysis; (**c**) Kaplan–Meier curve showing difference in survival between control group (light purple line) and supplement group (dark purple line). The log-rank test was used for statistical analysis.

The mortality rate of dogs in the control group (80%) was higher than that of the supplement group (30%) (Figure 1a,b). The Kaplan–Meier curve was used to demonstrate the survival time from a certain date to the time of IMHA dog death (Figure 1c). The results show that the killing ability of IMHA was time-dependent. In the control group, the survival time was 11 post-therapeutic days and the survival rate markedly declined to 16.7 ± 10.7% at 28-day post-therapeutic observation, whereas the survival rate of dogs in the supplement group was 66.7 ± 19.2% throughout the study period (log-rank test, *p* = 0.408).

### 3.4. Post-Therapeutic Clinical Pathology

**Figure 2:**
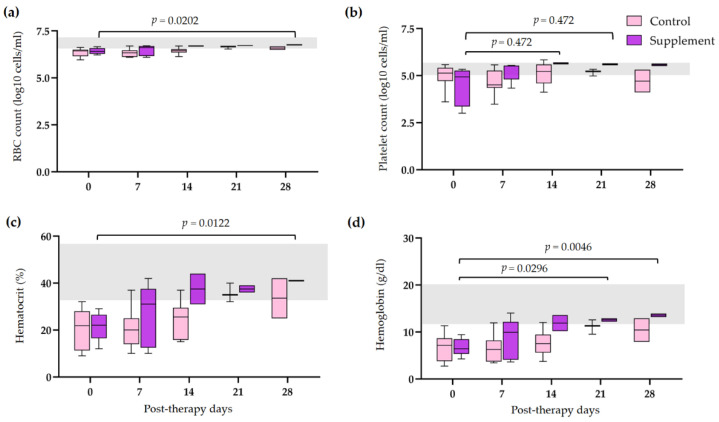
Comparison of erythrogram and platelet count in IMHA dogs treated with prednisolone alone and prednisolone combined with EAB-277 at five different time intervals (0, 7, 14, 21, and 28 days). Box and whisker plot showing (**a**) red blood cell count, (**b**) platelet count, (**c**) hematocrit, (**d**) hemoglobin of the control group (purple boxes) and the supplement group (pink boxes) with normal range reference (grey bar). The Mann–Whitney U test or the Wilcoxon signed rank test was used for statistical analysis. Horizontal lines with *p* values illustrate the comparisons between treatment groups at each end of the line.

**Figure 3:**
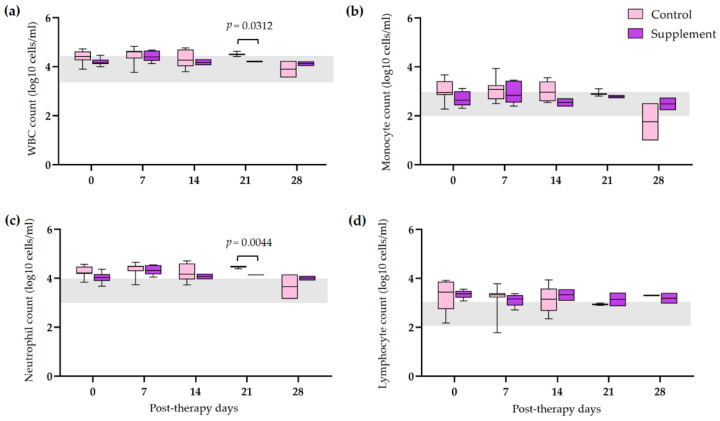
Comparison of leukogram in IMHA dogs treated with prednisolone alone and prednisolone combined with EAB-277 at 5 different time intervals (0, 7, 14, 21 and 28 days). Box and Whisker plot showing (**a**) total white blood cell count, (**b**) monocyte count, (**c**) neutrophil count, (**d**) lymphocyte count of the control group (purple boxes) and the supplement group (pink boxes) with normal range reference (grey bar). The Mann–Whitney U test or the Wilcoxon signed rank test was used for statistical analysis. Horizontal lines with *p* values illustrate the comparisons between treatment groups at each end of the line.

**Figure 4:**
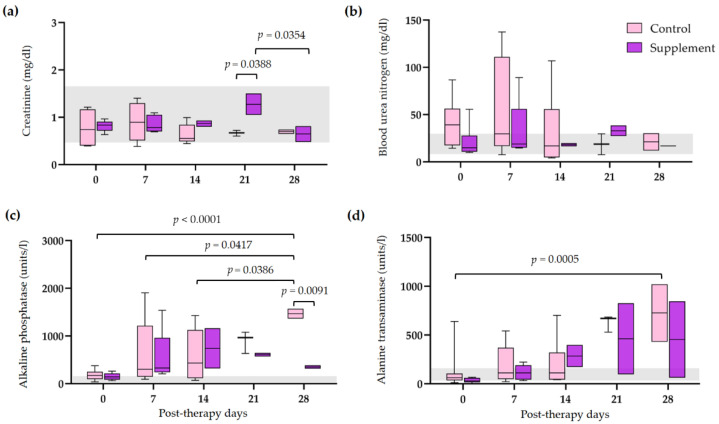
Comparison of serum biochemical profiles in IMHA dogs treated with prednisolone alone and prednisolone combined with EAB-277 at 5 different time intervals (0, 7, 14, 21, and 28 days). Box and whisker plot showing (**a**) creatinine, (**b**) blood urea nitrogen, (**c**) alkaline phosphatase and (**d**) alanine transaminase of the control group (purple boxes) and the supplement group (pink boxes) with normal range reference (grey bar). The Mann–Whitney U test or the Wilcoxon signed rank test was used for statistical analysis. Horizontal lines with *p* values illustrate the comparisons between treatment groups at each end of the line.

The hematological and blood chemistry parameters in IMHA dogs treated with prednisolone alone and prednisolone combined with EAB-277 at five different time intervals (0, 7, 14, 21, and 28 days) were compared. There was no difference in the red blood cell (RBC) count, Hct, Hb, and platelet count between the control group and the supplement group. However, all erythrocyte parameters were statistically increased from baseline to 28 post-therapeutic days in the supplement group (*p* < 0.05) (Figure 2a,b,d). The platelet count of the supplement group was also statistically increased from baseline, 14, and 21 post-therapeutic days (*p* < 0.05) (Figure 2c). No differences in leukocyte parameters within each group were found throughout the trial. Nevertheless, total WBC and neutrophil counts in the supplement group were lower than that of the control group at 21 post-therapeutic days (*p* < 0.05) (Figure 3a,c). The creatinine (Cr) levels of both groups remained within the normal range throughout the duration of the investigation (Figure 4a). Both groups had also the same amounts of blood urea nitrogen (BUN) (Figure 4b). Serum alkaline phosphatase (ALP) levels in the control group were higher at 28 post-therapeutic days than at 0, 7, 14, and 21 post-therapeutic days, whereas there was also difference between the control and supplement group during the 28 post-therapeutic days (*p* < 0.05) (Figure 4c). Only the control group’s serum alanine transferase (ALT) increased from its baseline level at 28 post-therapeutic days (*p* < 0.05) (Figure 4d).

### 3.5. Tapering of Immunosuppressive Therapy

**Figure 5:**
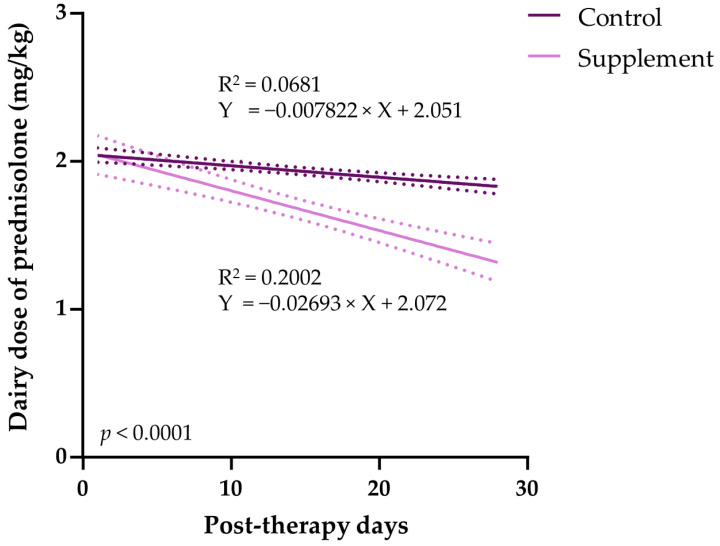
Comparison of the daily doses of prednisolone in IMHA dogs treated with prednisolone alone (control group) and prednisolone combined with EAB-277 (supplement group) for 28 days. Linear regression analysis obtained comparing the daily prednisolone dosages in IMHA dogs treated with prednisolone alone (control group, dark purple line) and prednisolone combined with EAB-277 (supplement group, light purple line). The green lines represent the 95% upper and lower confidence levels.

All dogs with IMHA were treated with an immunosuppressive dose of prednisolone (median PO dose, 2.0 mg/kg q12 h) at the time of presentation. In the supplement group, the dogs with IMHA were orally treated with EAB-277 for 28 days with an initial dosage of 100 mg/10 kg and a maintenance dosage of 50 mg/10 kg. The tapering of the initial prednisolone dose was carried out at the point of clinical improvement and stable Hct. The results demonstrate that the number of dose-tapered animals in the control group (2/12 animals) was less than the supplement group (3/6 animals). Additionally, the incidence density, the new tapering per 100 animal-day at opportunity, in the control group (0.63) was lower than that of in the control group (2.68). Therefore, the EAB-277-treated group showed a marked reduction in the number of patients with tapered dosages, with a 2.05 incidence rate difference. Moreover, the incidence rate ratio (IRR) indicates that the dogs supplemented with EAB-277 had 4.20 times the tapering rate of the immunosuppressive agent compared to the control group during the study period (Table 2). After tapering, the immunosuppressive dosage, the size of daily dosage, was measured and the linear regression analysis was used to evaluate whether EAB-277 influenced the post-therapeutic duration of prednisolone dose-tapering. The results show that the tapering of prednisolone correlated negatively with post-therapeutic duration in the control group (*R*^2^ = 0.0681) and in the supplement group (*R*^2^ = 0.2002). The slopes of the control (0.0078, 95%CI = 0.0109–0.0047) and the supplement group (0.0269, 95%CI = 0.0352–0.0187) differed significantly (*p* < 0.0001 using two-tailed Student’s *t*-test) (Figure 5). Thus, EAB-277 affects the post-therapeutic dependence of prednisolone dose-tapering in this study.

## 4. Discussion

The lipid fraction extracted from greenshell mussels is highly significant in both human and veterinary medicine. A detailed chemical analysis of the PCSO-524^®^ has been previously published [19,20]. The blend of greenshell mussel oil includes up to 91 fatty acids, with 16 accounting for more than 1% of the total fatty acid. There were 17 saturated fatty acids found, with the highest concentrations were of 14:0 myristic acid, 16:0 palmitic acid, and 18:0 steroic acid. Among the 10 n-3 PUFAs found were 20:5n3 EPA and 22:6n3 DHA [11]. These findings are in agreement with our results on the EAB-277 derived greenshell mussels and krill, the major constituents of which were C18:1n9c oleic acid, C16:0 palmitic acid, C20:5n3 EPA, C18:2n6c linolelaidic acid, and 22:6n3 DHA, respectively. Thus, this EAB-277 product was developed from PCSO-524^®^, a lipid fraction derived from greenshell mussels coupled with krill oil extract. The krill oil extract has high levels of fatty acids including C16:0 palmitic acid, C16:1 Palmitoleic acid, C18:1 Oleic acid, C20:5 EPA, C14:0 myristic acid, and C22:6 DHA [21,22]. Consequentially, the EAB-277 has demonstrated a significant amount of anti-inflammatory EPA and DHA. However, these bioactive molecules could be affected by ecosystem alterations caused by seasonal and climatic temperature. Therefore, a minor (10%) variation in the saturated, monounsaturated, and PUFA content throughout the manufacturing process is considered acceptable [23]. Unfortunately, there are no studies or specific information regarding the pharmacokinetic and bioavailability of EAB-277 in dogs, making it difficult to estimate the appropriate dose of the nutraceutical therapeutic adjuvant.

The present study provides evidence for the advantageous outcomes of EAB-277 supplementation in conjunction with standard therapy for dogs diagnosed with IMHA. Firstly, no differences in all variables were confirmed between IMHA dogs in the control and supplement groups at the initial clinical presentation. The major findings of this study are as follows. According to the Kaplan–Meier curve, EAB-277 supplementation for 28 days led to a tendency towards a greater survival rate as compared to the control group. However, statistical analysis results indicated that this difference was not statistically significant. These findings are consistent with prior research, which found that the majority of deaths occurred within the first two weeks [24]. The risk of mortality associated with a hemolytic component value was previously documented [25]. With the overall quantity of erythrocyte destruction, a dog develops severe anemia, which is potentially life-threatening. It is noteworthy that a comparison between pre-therapy and post-therapy measurements reveals a decrease in hemolysis subsequent to the administration of EAB-277, as indicated by the enhancement in RBC, Hb, and Hct parameters. Additionally, findings of the current research reveal a considerable increase in platelet counts within the supplemented group. Regrettably, no significant differences were observed between the supplemented and control groups. In agreement with previously reported data, the mean estimated risk of mortality in dogs with IMHA reduced by 16% for each unit rebound in plasma mean platelet component concentration [26]. Thus, the platelet count may be helpful for predicting prognosis and treatment monitoring in dogs with IMHA.

IMHA is a severe systemic inflammation that frequently results in neutrophilia and an increase in serum neutrophil extracellular traps [27]. Interestingly, the leukocyte parameters in the IMHA dogs were affected by the 21-day EAB-277 supplementation. Additionally, the EAB-277 supplementation decreased the inflammatory response, as demonstrated by reductions in total WBC and neutrophil counts. Surprisingly, the previous research additionally demonstrated that EAB-277 supplementation could reduce levels of inflammatory cytokines including TNF-α and IL-6 as well as malondialdehyde in dogs with tracheal collapse [28]. In human autoimmune disease models, overproduction of the IL-6 and TNF-α cytokines has been linked to develop spontaneous autoimmune diseases; this might be due to their potential to reduce the suppressive activity of Tregs [29,30]. Moreover, a prior study also revealed that IMHA dogs had greater levels of serum pro-inflammatory cytokines (IL-2, IL-6, CXCL-8, and TNF-α) than healthy dogs [31]. Since then, the effects of marine PUFAs on the COX and LOX pathways have been well characterized. As a consequence, its application involves a variety of inflammatory and degenerative conditions [13,32].

Prednisolone dose-tapering and IRR in this research was affected by post-therapeutic dependency on EAB-277, although greater prednisolone doses might increase the risk and severity of side effects even if the prednisolone fractionated regimen exhibited a delayed amelioration of adverse effects [33]. Several studies have documented prednisolone therapy’s adverse effects [34,35,36]. Moreover, the quality of life of an animal may be affected by adverse effects from therapy, as well as owner compliance and willingness to continue treatment [37]. With regard to blood chemistry, these findings indicate that the use of EAB-277 helps to decrease the adverse reactions of immunosuppressive therapy in IMHA dogs. Additionally, the EAB-277 supplementation helped IMHA dogs maintain consistent serum enzymes. In contrast, the levels of serum ALT and ALP in the control group were significantly higher at 28 post-therapeutic days compared to baseline. The available literature suggests that the precise pathophysiological mechanisms underlying the development of hepatic hypoxia in IMHA remain unclear. Nevertheless, some studies propose a potential link between elevated levels of liver enzymes, including ALP and AST, and hepatic hypoxia [38]. Hence, it is plausible that the observed decrease in hepatic enzyme levels in the context of this current inquiry may stem from both the improvement of the underlying IMHA pathology in dogs and the tapering of prednisolone dosages. These findings are also in accordance with a previous study on ALP and ALT parameters in dogs with tracheal collapse who received EAB-277 supplementation for 5 weeks [28]. Furthermore, the PCSO-524 supplementation had no adverse effects on blood profile parameters, which is consistent with prior research in healthy beagle dogs [39].

The primary limitation of this study is the small sample size employed. To corroborate the present findings, it is recommended to conduct future research involving larger sample sizes and longer observation periods. Additional inquiries into the advantageous impact of intervention on treatment outcomes may also be necessary. The absence of statistical significance observed may be attributed to the low statistical power resulting from the limited number of cases included in the study. For limitation of non-blind experimental grouping, this can lead to performance-biased outcomes. However, as this testing was executed in an open-label format, deliberate bias was less likely to occur due to the adherence of the ACVIM consensus statement criteria for prednisolone dose-tapering utilized during the course of the study [10]. In addition, conducting an in-depth examination of other pertinent clinicopathological characteristics and prognostic markers may facilitate the understanding of the precise mechanisms underlying the purported benefits of EAB-277 intervention for dogs affected by IMHA.

## 5. Conclusions

Standard therapy supplemented with EAB-277 could improve hematological and blood chemistry profiles, resulting in a tendency towards an increased survival rate in dogs with IMHA. Moreover, EAB-277 supplementation can also attenuate prednisolone dose-tapering and increase the quality of life of IMHA dogs after 28 days of medication. However, a longer-term investigation with a larger sample size is required to confirm these outcomes.

## Figures and Tables

**Table 1:** Comparison of variables between IMHA dogs within the control group and supplement group at the time of diagnosis.

	Control Group	Supplement Group	*p*-Value *
Weight ^†^ (kg)	11.15 (5.00–27.65)	7 (3.55–17.20)	0.2767
Age ^†^ (year)	9 (2.7–11.1)	8.9 (2.3–11.1)	0.8589
Sex	Male 6 Female 6	Male 2 Female 4	0.6380
Hct ^‡^ (%)	20.00 ± 8.22	21.40 ± 6.23	0.7065
Hb ^‡^ (g/dL)	6.58 ± 2.66	6.85 ± 1.84	0.8133
Spherocytosis ^#^	2/12	4/6	0.1070
Positive ROFT ^#^	12/12	6/6	>0.9999
Positive saline agglutination test ^#^	2/12	3/6	0.2682
Positive DAT ^#^	12/12	6/6	>0.9999

^†^ values listed as median and (range); ^‡^ values listed as mean ± standard deviation; ^#^ number of affected or positive dogs per number of performed dogs. * Wilcoxon’s signed ranks test or Fisher’s exact test. Abbreviations: Hct, hematocrit; Hb, hemoglobin; ROFT, rapid osmotic fragility test; DAT, direct antiglobulin test.

**Table 2:** Comparison of incidence density of prednisolone tapering between IMHA dogs within the control group and supplement group.

	Control	Supplement
Eligible animals for tapering	12.00	6.00
Tapered animals	2.00	3.00
Animal–day at opportunity for tapering	315.00	112.00
New tapering per 100 animal–day at opportunity	0.63	2.68
Incidence rate difference		2.05
Incidence rate ratio		4.2

## Data Availability

The data supporting this study are available from the corresponding author upon reasonable request.
